# Annotations

**Published:** 1899-09-30

**Authors:** 


### Annotations.

### Colour-blind Sailors.

We cannot but feel that Mr. Bickerton, in his address at Portsmouth on colour-blindness and defective sight in the mercantile marine, made good the indictment which he brought against the Board of Trade of something worse than mere supineness in regard to this important question. It seems so reasonable a suggestion, and one so in accordance with common sense, that sailors—above all people in the world—should possess good sight, seeing how absolutely the safety of all ships depends on the accurate eyesight of those on look-out duty, that we can hardly avoid a feeling of indignation at the indifference to the subject shown by the great public department which has the control of our mercantile marine. No check of any kind seems yet to have been placed upon the entrance of colour-blind or poor-sighted boys to the sea life, so that there are constantly among our seamen a large number of colour-blind and nautically-blind men who may continue in the service to the end of their days. Truly when sailors rise in the ranks and apply for officers' certificates they have to undergo an examination, and if colour-blind, or if nautically blind (*i.e.*, if they possess less than one-half the normal vision), these facts are endorsed on their certificates; but, according to Mr. Bickerton, even when these conditions are discovered, persons so affected are still "permitted without let or hindrance to follow the sea." Plenty of reasons are given for believing that many serious collisions and other accidents have happened in consequence of defective vision in the "look-out"; but a further charge is brought against the Board of Trade, namely, that while they maintain that there is no evidence to connect shipping disasters with defective eyesight, they decline to have the eyesight of the witnesses examined when the causes of these disasters are being inquired into. Never was there a clearer case of a department burying its head in the sands of red tape and officialdom.

### Self-conceit.

So well-recognised is it that in many cases exaggerated self-assurance is a symptom of definite brain disease that it is perhaps as well to be reminded, as we are by Dr. Harry Campbell's remarks before the British Medical Association, of the amount of self-assurance which a man may display even though in other respects he may be sane. We all know the sort of person who is swollen up with self-importance, who in everything he does "puts on a lot of side," and often, by mere force of "bounce" gets his own way. It is, indeed, to many a humble-minded man a matter of no small distress, and even of still further humiliation, to see himself over-topped in all he does by men inferior to himself in everything but self-assurance. It is self-assurance, what in slang phrase is described as "bounce," or "cheek," or "side" which helps many people to the front. Hence this is an attribute of much utility. In some cases, however, so excessive is it that it may properly be termed morbid, but even then, as Dr. Harry Campbell points out, it may not be pathological. As an instance he refers to the case of a man who exhibits so large a degree of self-importance that, if he had not convincing proof to the contrary, he would unhesitatingly set him down as a general paralytic. "He holds up his chin, pouts out his chest and struts about . . . the very embodiment of self-conceit. I have," he says, "seen a crowd actually divide to make a lane for him, while all eyes gazed upon him in mingled wonder and amusement, as he strutted serenely on. . . . Yet he is, as a matter of fact, by no means wanting either intellectually or physically . . . he is simply the victim of overweening and irrepressible self-conceit." But there are many examples and degrees of the same peculiarity, as in the Parliamentary bore, or the dreary after-dinner speaker; even the meetings of our learned societies are by no means exempt from exhibitions of a form of self-assurance which is morbid without being pathological. In some cases, however, especially where this sort of thing is seen to develop itself in the autumn of life, and to intrude itself as a new mental trait, we find ourselves on the borderland, and it may be very difficult to be sure that it is not an indication of insanity. In regard to some cases of this kind Dr. Harry Campbell makes the not inapt suggestion that they may really be cases of abortive general paralysis, a disease which, as is now well recognised, does not always pass beyond its incipient stage, remaining stationary for years, and perhaps never going further. In any case, however, it must be remembered that, although morbid self-assurance may not always be pathological, its occurrence as a new feature in a man's mental life must always be regarded with great suspicion, general paralysis of the insane being so often ushered in by this very symptom.

### The Tragedy at Eastbourne.

The sad death of Dr. Dick at Eastbourne is a matter for great regret, but it teaches several morals. First of all, of course, is the urgent necessity of taking every possible precaution to distinguish between dispensing bottles containing poisonous drugs and those whose contents are of an innocuous nature, or are usually employed in large doses. Next comes the important lesson that one should always be ready to recognise the possibility of one's own fallibility. The tragedy at Eastbourne is but a replica of what has happened again and again in various parts of the world. Complaint is made by a patient that there is something wrong with the medicine; the doctor, absolutely convinced that the medicine is right, ridicules the suggestion, and to convince the patient there and then drinks a large dose; and afterwards, too late, it turns out that from some accidental cause the medicine was wrong after all. The possibility of error in dispensing should be the first thought, rather than the last, whenever a medicine is said to disagree. But it is not so, and case after case has occurred in which a doctor has sacrificed himself rather than admit that he could have erred in so simple a matter, whereas it really is in the most simple, the almost automatic actions that error most easily creeps in. This unfortunate medical man, even to the last, asserted that he never put strychnine into the medicine, and implored those around to solve the mystery. Yet this does not seem to have been so very difficult a matter. The bottles of chloroform water and strychnine solution were standing near one another, though on different shelves, and were very similar, while that containing the strychnine was not labelled poison. The probability is that, instead of chloroform water, a couple of ounces of strychnine solution were put into the medicine. Hence the tragedy. It is very sad. Let us extract from it the warning.

